# Supplementary material for: RudS: bacterial desulfidase responsible for tRNA 4-thiouridine de-modification
Source: Nucleic Acids Res. 2024 Aug 21;52(17):10543–62. doi: 10.1093/nar/gkae716 (PMC11417400; doi:10.1093/nar/gkae716)
Supplement: gkae716_Supplemental_Files [file gkae716_supplemental_files.zip › RudS_Supplement.pdf]

RudS: Bacterial Desulfidase Responsible for tRNA 4-Thiouridine De-modification

Rapolas Jamontas[1], Audrius Laurynėnas[2], Deimantė Povilaitytė[1], Rolandas Meškys[1], Agota Aučynaitė[1]*

[1] Department of Molecular Microbiology and Biotechnology, Institute of Biochemistry, Life Sciences Center, Vilnius University, 10257 Vilnius, Lithuania

[2] Department of Bioanalysis, Institute of Biochemistry, Life Sciences Center, Vilnius University, 10257 Vilnius, Lithuania

*To whom correspondence should be addressed. Tel: +37062082954; Email: agota.aucynaite@bchi.vu.lt

SUPPORTING INFORMATION

MATERIAL AND METHODS

Supplementary Table 1. Bacterial strains used in this study.

Bacterial strain	Comments	Source
Escherichia coli DH5α	Used for routine DNA manipulations	Pharmacia, USA
Escherichia coli BL21(DE3)	Used for recombinant protein overexpression	Avidis, France
Pseudomonas sp. MIL9	NCBI accession ID: PRJNA698458	Isolated from soil; Vilnius university Life Sciences Center (1)
Pseudomonas sp. MIL19	NCBI accession ID: PRJNA877084	Isolated from soil; Vilnius university Life Sciences Center
Pseudomonas putida KT2440	DSM No.: 6125	Leibniz Institute DSMZ-German Collection of Microorganisms and Cell Cultures GmbH
Salmonella enterica subsp. *enterica* serovar Typhimurium LT2 Δ*pyrF*	Uracil auxotroph	Kind gift from Gunilla Jäger, Umeå University, Sweden
Thermus thermophilus HB8	DSM No.: 579	Leibniz Institute DSMZ-German Collection of Microorganisms and Cell Cultures GmbH

Supplementary Table 2. Plasmid vectors used in this study.

Plasmid vector	Comments	Source
pLATE11	Empty vector constructed by amplification of backbone and blunt-end ligation.	Thermo Fisher Scientific, USA and this study
pLATE11-TudS	TudS encoding expression vector	(2)
pLATE11-YbgA	DUF1722 encoding expression vector	This study
pBR322-RudS_vir	Synthetic vector encoding viral RudS	Synthesized by Thermo Fisher Scientific, USA
pLATE11-RudS_KT	RudS encoding expression vectors	This study
pLATE11-RudS_ST		
pLATE11-RudS_PP		
pLATE11-RudS_TT		
pLATE11-RudS_vir		
pLATE11-RudS_PU		
pLATE52-RudS_KT	RudS with N-terminal 6xHis-tag encoding expression vectors	This study
pLATE52-RudS_ST		
pLATE52-RudS_PP		
pLATE52-RudS_TT		
pLATE52-RudS_vir		
pLATE52-RudS_PU		
pLATE52-YbgA	DUF1722 with N-terminal 6xHis-tag encoding expression vectors	This study
pLATE11-RudS_KT_C114A	RudS with conservative cysteine substitution encoding expression vectors	This study
pLATE11-RudS_KT_C49A		
pLATE11-RudS_KT_C17A		
pLATE11-RudS_ST_C12A		
pLATE11-RudS_ST_C44A		
pLATE11-RudS_ST_C110A		
pLATE11-RudS_PP_C18A		
pLATE11-RudS_PP_C50A		
pLATE11-RudS_PP_C115A		
pLATE11-RudS_TT_C16A		
pLATE11-RudS_TT_C47A		
pLATE11-RudS_TT_C111A		
pLATE11-RudS_vir_C12A		
pLATE11-RudS_vir_C44A		
pLATE11-RudS_vir_C110A		
pLATE11-RudS_PU_C15A		
pLATE11-RudS_PU_C47A		
pLATE11-RudS_PU_C112A		
pLATE52-RudS_KT_C114A	RudS_KT with conservative cysteine substitution and N-terminal 6xHis-tag encoding expression vectors	This study
pLATE52-RudS_KT_C49A		
pLATE52-RudS_KT_C17A		
pLATE11-RudS_KT_R24M	RudS_KT with amino acid predicted to participate in substrate binding and/or catalysis substitution and encoding expression vectors	This study
pLATE11-RudS_KT_Y25M		
pLATE11-RudS_KT_Y25A		
pLATE11-RudS_KT_Y25F		
pLATE11-RudS_KT_N26M		

pLATE11-RudS_KT_G27M		
pLATE11-RudS_KT_H29M		
pLATE11-RudS_KT_K30M		
pLATE11-RudS_KT_D33M		
pLATE11-RudS_KT_R36M		
pLATE11-RudS_KT_K37M		
pLATE11-RudS_KT_E51M		
pLATE11-RudS_KT_R60M		
pLATE11-RudS_KT_D61M		
pLATE11-RudS_KT_R64M		
pLATE11-RudS_KT_K110M		
pLATE11-RudS_KT_S111M		
pLATE11-RudS_KT_S113M		
pLATE11-RudS_KT_E117M		
pLATE11-RudS_KT_R118M		
pLATE11-RudS_KT_K120M		
pLATE11-RudS_KT_Y122M		
pLATE11-RudS_KT_H127M		
pLATE11-RudS_KT_H131M		
pLATE11-RudS_KT_E152M		
pLATE11-RudS_KT_R155M		
pLATE11-RudS_KT_H157M		
pLATE11-RudS_KT_N202M		
pLATE11-RudS_KT_N203M		
pLATE11-RudS_KT_Q205M		
pLATE11-RudS_KT_R240M		
pLATE11-RudS_KT_C241M		
pLATE11-RudS_KT_S243M		
pLATE11-RudS_KT_R244M		
pLATE11-RudS_KT_T246M		
pLATE52-RudS_KT_R24M	RudS with amino acid predicted to participate in substrate binding and/or catalysis substitution and N-terminal 6xHis-tag encoding expression vectors	This study
pLATE52-RudS_KT_N26M		
pLATE52-RudS_KT_G27M		
pLATE52-RudS_KT_H29M		
pLATE52-RudS_KT_K30M		
pLATE52-RudS_KT_E51M		
pLATE52-RudS_KT_R60M		
pLATE52-RudS_KT_R64M		
pLATE52-RudS_KT_K110M		
pLATE52-RudS_KT_S111M		
pLATE52-RudS_KT_S113M		
pLATE52-RudS_KT_R118M		
pLATE52-RudS_KT_K120M		
pLATE52-RudS_KT_Y122M		
pLATE52-RudS_KT_E152M		
pLATE52-RudS_KT_R155M		
pLATE52-RudS_KT_H157M		
pLATE52-RudS_KT_N202M		
pLATE52-RudS_KT_N203M		
pLATE52-RudS_KT_R240M		

pLATE52-RudS_KT_C241M		
pLATE52-RudS_KT_S243M		
pLATE52-RudS_KT_R244M		
pLATE52-RudS_KT_T246M		

Supplementary Table 3. Oligonucleotide primers used in this study.

Primer name	Sequence (>3')	Purpose
pLATE11_empty_FW	TGACTTCCCATCTCCGGTTT	Amplification of vector backbone for empty vector creation.
pLATE11_empty_RV	AGTTATATCTCCTTCTGGATTTAAATGTTA	
RudS_KT_52_FW	GGTTGGGAATTGCAACACGACCCTTCCGCCCAC	Amplification of *rudS_KT* gene for cloning into pLATE11 and pLATE52 vectors
RudS_KT _11_FW	AGAAGGAGATATAACTATGCACGACCCTTCCGCC	
RudS_KT _11_52_RV	GGAGATGGGAAGTCATCAGACCGCATTGCGCAAG	
RudS_ST_11_31_FW	AGAAGGAGATATAACTATGAATAACAAACCCGTAGTCGGC	Amplification of *rudS_ST* gene for cloning into pLATE11 and pLATE52 vectors
RudS_ST_11_52_RV	GGAGATGGGAAGTCATTAGGTGATGGCTAAGCGCAG	
RudS_ST_52_FW	GGTTGGGAATTGCAAAATAACAAACCCGTAGTCGGC	
RudS_PP_11_31_FW	AGAAGGAGATATAACTATGCGCTCACCAGAAACCATCACC	Amplification of *rudS_PP* gene for cloning into pLATE11 and pLATE52 vectors
RudS_PP _11_52_RV	GGAGATGGGAAGTCATTATAGGGCGTTACGCAAGCTG	
RudS_PP _52_FW	GGTTGGGAATTGCAACGCTCACCAGAAACCATCACC	
RudS_TT_11_31_FW	AGAAGGAGATATAACTATGAGCCCAGGGGCGTGG	Amplification of *rudS_TT* gene for cloning into pLATE11 and pLATE52 vectors
RudS_TT_52_FW	GGTTGGGAATTGCAAAGCCCAGGGGCGTGG	
RudS_TT_11_52_RV	GGAGATGGGAAGTCATTAGGCCCCGGGGACGTG	
RudS_PU_11_31_FW	AGAAGGAGATATAACTATGCTCCCGTCCCCTGCCAAAC	Amplification of *rudS_PU* gene for cloning into pLATE11 and pLATE52 vectors
RudS_PU_11_52_RV	GGAGATGGGAAGTCATTAGATGGCGTTTCGCAGGCTG	
RudS_PU_52_FW	GGTTGGGAATTGCAACTCCCGTCCCCTGCCAAAC	
RudS_vir_11_52_RV	GGAGATGGGAAGTCATTAGTTCACCAAGAGTCTTAGAGCCAGTT CATC	Amplification of *rudS_vir* gene for cloning into pLATE11 and pLATE52 vectors
RudS_vir_11_31_FW	AGAAGGAGATATAACTATGATAAAAAAACCTGTCGTTGGGATCA GTGG	
RudS_vir_52_FW	GGTTGGGAATTGCAAATAAAAAAACCTGTCGTTGGGATCAGTGG	
YbgA_11_31_FW	AGAAGGAGATATAACTATGAATCTACAACGATTTGATGACAG	Amplification of *ybgA* gene for cloning into pLATE11 and pLATE52 vectors.
YbgA_11_52_RV	GGAGATGGGAAGTCATTATAAAACTCCTGAATGGCGC	
YbgA_52_FW	GGTTGGGAATTGCAAAATCTACAACGATTTGATGACAG	

RudS_ST_C12A	AGTCGGCATTAGCGGAGCTCTGACTGGCGCC	Site directed mutagenesis of conservative cysteines using pLATE11 or pLATE52 vectors with insert as a template.
RudS_ST_C44A	CCTACAAACCGATCGCCCCGGAGGTGGCGA	
RudS_ST_C110A	TGTGTGTGCAAAATCGCCCAGTGCCGGAATGGAGCG	
RudS_TT_C16A	TGTGGTGAGCGCCGCCCTGGGGTTCGCC	
RudS_TT_C47A	CTTCGTCCCCGTCGCCCCGGAGGTGGAG	
RudS_TT_C111A	CCGCTCCCCCTCCGCCGCCCTGAAGGAC	
RudS_KT_C17A	CGCCATCAGCGCCGCCCTGACCGGGCAC	
RudS_KT_C49A	GACTGGCTACCCGTGGCTCCGGAAGTGGCAAT	
RudS_KT_C114A	TCTTCATGCAGAAGTCACCTTCAGCCGGCCTGGAACG	
RudS_PP_C18A	TGGGCATCAGTGCCGCCTTACTGGGCTCCG	
RudS_PP_C50A	TTTCGCCCCCGTGGCCCCCGAAGTAGGG	
RudS_PP_C115A	CAGTCCCCGTCCGCCGGCCTGCACCG	
RudS_PU_C15A	CGCCATCAGCGCCGCCCTGATGGGCGCA	
RudS_PU_C47A	TTTCGTGCCGGTCGCCCCGGAAGTCGCG	
RudS_PU_C112A	GAAATCGCCGTCCGCCGGACTGGAGCGG	
RudS_vir_C12A	TCGTTGGGATCAGTGGTGCTTTGGCCGGTTCTTCTG	
RudS_vir_C44A	TGGAATGGGTAACATTCAAACCAGTAGCTCCGGAAATGGCTA	
RudS_vir_C110A	GTGCAAAATCTCCTAGCGCTGGTATGGAACGCGTGC	
RudS_KT_R24M	ACCGGGCACAGCGTGATGTACAACGGCGGCCAC	Site directed mutagenesis of residues predicted to participate in catalysis or/and substrate binding using pLATE11 or pLATE52 vectors with insert as a template.
RudS_KT_Y25A	GTGGCCGCCGTTGGCGCGCACGCTGTGC	
RudS_KT_Y25F	GGCCGCCGTTGAAGCGCACGCTG	
RudS_KT_Y25M	CTTGTGGCCGCCGTTCATGCGCACGCTGTGCCC	
RudS_KT_N26M	GTGGCCGCCCATGTAGCGCACGCTGTGC	
RudS_KT_G27M	GGAGGCCTTGTGGCCCATGTTGTAGCGCACGCT	
RudS_KT_H29M	CAGGTCGGAGGCCTTCATGCCGCCGTTGTAGCG	
RudS_KT_K30M	CGGAGGCCATGTGGCCGCCGTTGT	
RudS_KT_D33M	GCTGTTTACGGCACAGCATGGAGGCCTTGTGGCCG	
RudS_KT_R36M	AAGGCCTCCGACCTGTGCATGAAACAGCTGGAACAGCAC	
RudS_KT_K37M	GTGCTGTTCCAGCTGCATACGGCACAGGTCGGAG	
RudS_KT_E51M	CCAAGCCGATTGCCACCATCGGACACACGGGTAGC	
RudS_KT_R60M	GGCTTGGGTGTCCCGATGGACCCGATTCGCCTG	
RudS_KT_D61M	GACCAGGCGAATCGGCATGCGCGGGACACCCAA	
RudS_KT_R64M	CCCGCGCGACCCGATTATGCTGGTCGGCAACCC	
RudS_KT_K110M	GGCCGCATGAAGGTGACATCTGCATGAAGATGTAG	
RudS_KT_S111M	CGTTCCAGGCCGCATGAAGGCATCTTCTGCATGAAGATGTAGC	
RudS_KT_S113M	CGTTCCAGGCCGCACATAGGTGACTTCTGCATGAAGATGTAGCC	
RudS_KT_E117M	CCTGATAAACCTTTACCCGCATCAGGCCGCATGAAGGTGAC	
RudS_KT_R118M	CCTGATAAACCTTTACCATTTCCAGGCCGCATGAAGGTGAC	
RudS_KT_K120M	TCCTGATAAACCATTACCCGTTCCAGGCCGC	
RudS_KT_Y122M	GGCCGTTGTCCTGCATAACCTTTACCCGTTCCAGGCCGC	
RudS_KT_H127M	TTATCAGGACAACGGCATGCCGGCCGTGCATGGTG	
RudS_KT_H131M	CCACCCGGCCGTGATGGGTGGCCGTGGCG	
RudS_KT_E152M	GCAGGCGGCCTTCCATTTCTACTGGCAGGTCCGGG	
RudS_KT_R155M	CCAGTAGAAGAAGAAGGCATGCTGCATGACCCTGTGCTG	
RudS_KT_H157M	GAAGAAGGCCGCCTGATGGACCCTGTGCTGCGC	
RudS_KT_N202M	CGATAGGCCTGGGGATTCATGGCCATCAGCAAATAC	
RudS_KT_N203M	GATAGGCCTGGGGCATGTTGGCCATCAGCAAATACTTGTA	

RudS_KT_Q205M	GATGGCCAACAATCCCATGGCCTATCGCACCCTC	
RudS_KT_R240M	GCAGGCCCTGCGCATGTGCGCCAGCCGCG	
RudS_KT_C241M	GCCGCGGCTGGCCATGCGGCGCAGGGC	
RudS_KT_S243M	GTGCCGCGCATGGCGCAGCGGCGCA	
RudS_KT_R244M	CGCCGCTGCGCCAGCATGGGCACCCACAGTAAC	
RudS_KT_T246M	GCCAGCCGCGGCATGCACAGTAACGTGC	

RESULTS

Supplementary table 4. Groups of amino acids that are close to tRNA and might play a role in enzymatic catalysis and/or substrate binding.

G1	G2	G3	G4	G5	G6
Arg24	Arg60	Lys110	Glu152	Asn202	Arg240
Tyr25	Asp61	Glu117	Arg155	Asn203	Cys241
Asn26	Arg64	Arg118	His157	Gln205	Ser243
Gly27		Lys120			Arg244
His29		Tyr122			Thr246
Lys30		His127			
Asp33		His131			
Arg36					
Lys37					

Detailed description of molecular dynamics simulations

The systems underwent minimization, heating to 300 K, and equilibration. Constant temperature and volume production runs (NTV) were conducted at 300 K using Langevin dynamics thermostat. For the initial simulations of the holoenzyme and complex, during heating and the first equilibration run of 200 ns, Cartesian positional restraints of 1 kcal/mol/$Å^2$ on backbone atoms of amino acids within ~7 Å around the iron-sulfur cluster were applied. These restraints were relaxed during the next 200 ns, and subsequent molecular dynamics simulations involved only distance restraints as specified. The same conditions were used for tRNA simulations.

The top HADOCK complex underwent a series of short molecular dynamics simulations, with 1 kcal/mol/ $Å^2$ restraints on protein backbone atoms and 5 kcal/mol restraints on the distance between the sulfur atom in the thiouridine moiety and the relevant iron atom. The distance restraint was incrementally reduced by one angstrom in each subsequent MD run until reaching a distance of 2 Å. The resultant complex then underwent 10 iterations of MD simulated annealing runs, each comprising 4 ns at temperatures ramping from 300 K to 400 K, 4 ns at 400 K, 4 ns cooling from 400 K to 250 K, 4 ns heating back to 350 K, 4 ns cooling back to 300 K, and finally 4 ns at 300 K, with restraints of 1 kcal/mol on protein backbone atoms and 5 kcal/mol/ $Å^2$ on the distance between the sulfur atom in the thiouridine moiety and the relevant iron atom. The final complex then underwent two different MD simulations, with the first one maintaining only 5 kcal/mol/ $Å^2$ restraint on the distance between the sulfur atom in the thiouridine moiety and the relevant iron atom, and the second one with an additional 4 distance restraints on specific Arg60, Glu51, Ser111, Ser113 atoms.

The best HDOCK model was used as a starting point in attempts to obtain a better model of the enzyme-substrate complex. Short molecular dynamics simulations with incrementally decreasing distance constraints for thiouridine sulfur and the relevant iron ion in the iron-sulfur cluster were conducted to bring the substrate and enzyme closer together. The obtained structures were then relaxed in 10 simulated annealing runs with previously described distance constraints and weak positional constraints on catalytic domain atoms within 7 angstroms around the iron-sulfur cluster. The resultant complex underwent initially restrained and restraint relaxation MD simulations as described in Methods and Materials, followed by a 640 ns simulation with the following 5 kcal/mol/ $Å^2$ distance constraints: Arg60 epsilon NH – Glu51 side chain carboxy O2; Glu51 side chain carboxy O1 – Ser113 side chain hydroxy group H; Ser113 side chain hydroxy group O – Ser111 side chain hydroxy group O, and thiouridine S – iron-sulfur cluster relevant iron ion. The last frame of this restrained simulation was used as an initial structure for two 260 ns simulations, where structure 1 retained the described above restraints, and structure 2 had only thiouridine S – iron-sulfur cluster restraint.

Supplementary Figure S1. Phylogenetic tree of 884 RudS homologues identified among complete reference genomes of 332 Archaea, 5042 Bacteria, and 47 viruses. The presence (green square) or absence of ThiI (red square) in the same genome is indicated. *Available for download as a separate supplementary material file.*

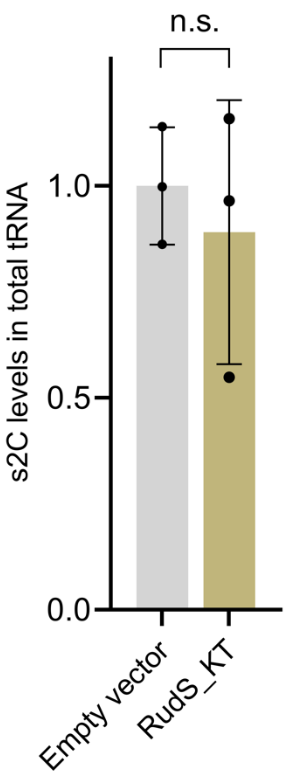

Supplementary Figure S2. s2C levels in *E. coli* total tRNA upon expression of RudS. n.s.: $p \geq 0.05$ compared to negative control (empty vector), Student's t-test.

.

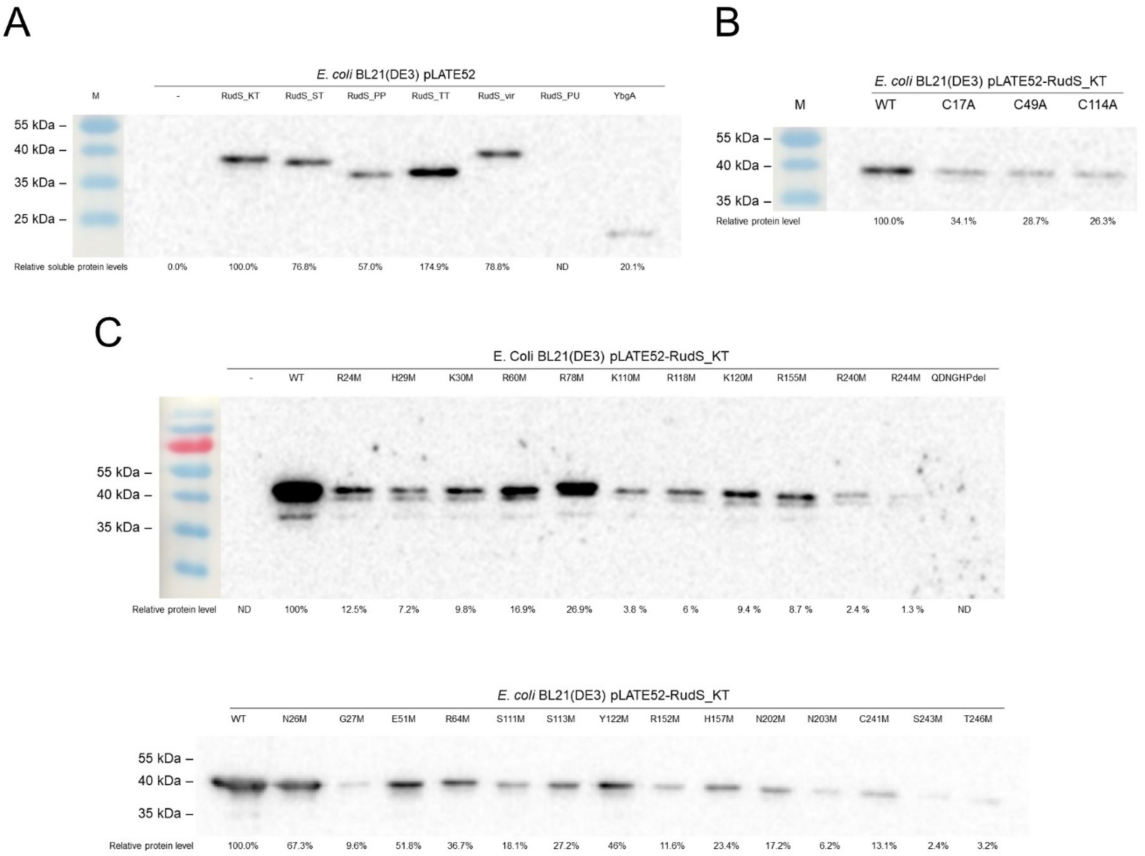

Supplementary Figure S3. Western blot analysis of soluble protein fractions in bacterial lysates (2 µg per lane). A: TudS-DUF1722 and DUF1722 (YbgA) domain containing proteins. B: RudS_KT mutants with substituted cysteines involved in iron-sulfur cluster formation. C: RudS_KT mutants having affected in vivo activity and predicted to participate in catalysis and/or substrate binding. Relative protein levels are normalized to wild type RudS_KT.

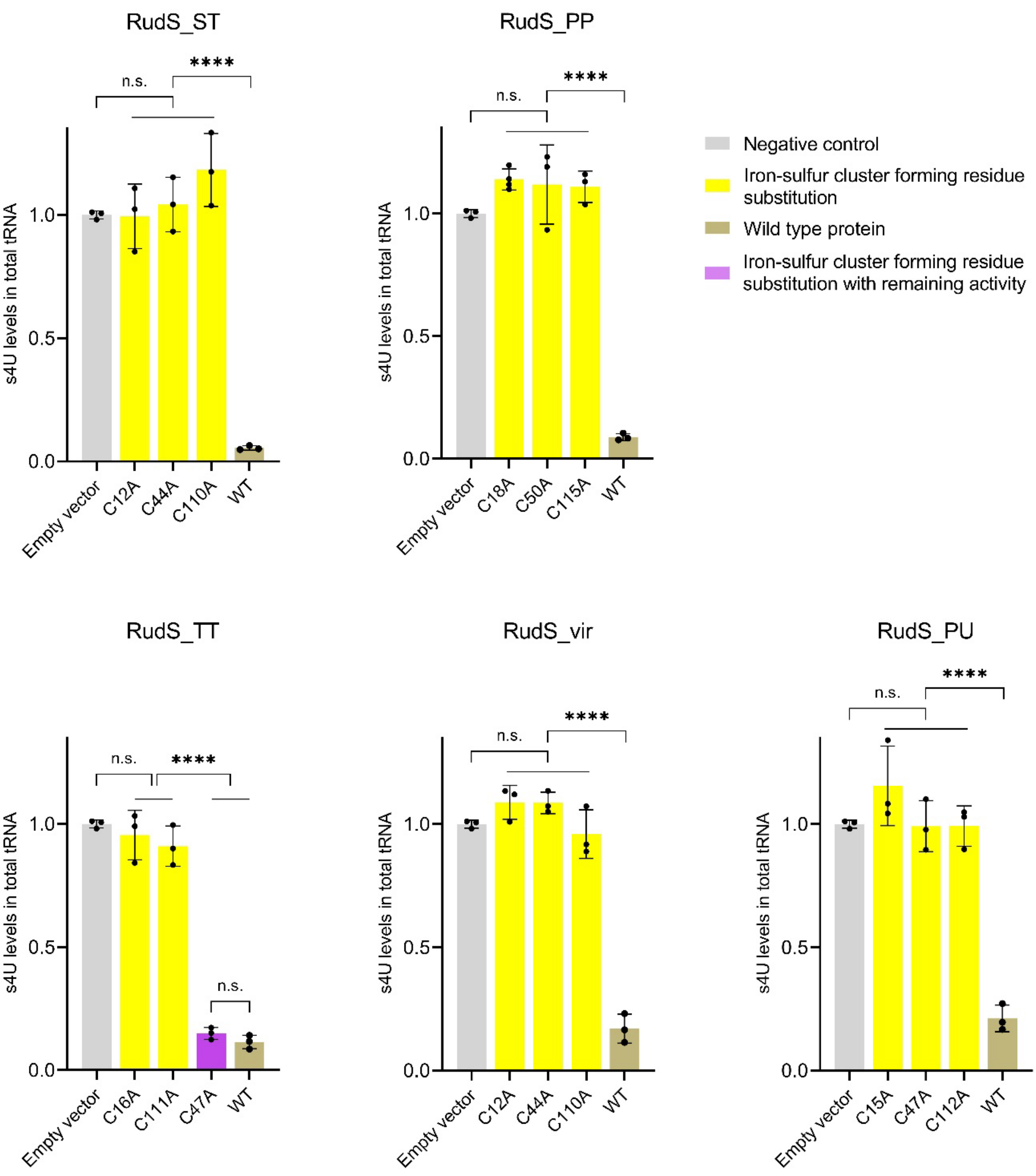

Supplementary Figure S4. s4U levels in *E. coli* total tRNA upon expression of RudS with conserved cysteine substitutions. ****: $p<0.0001$, n.s.: $p\geq0.05$ compared to WT, one way ANOVA with Bonferroni's post hoc test.

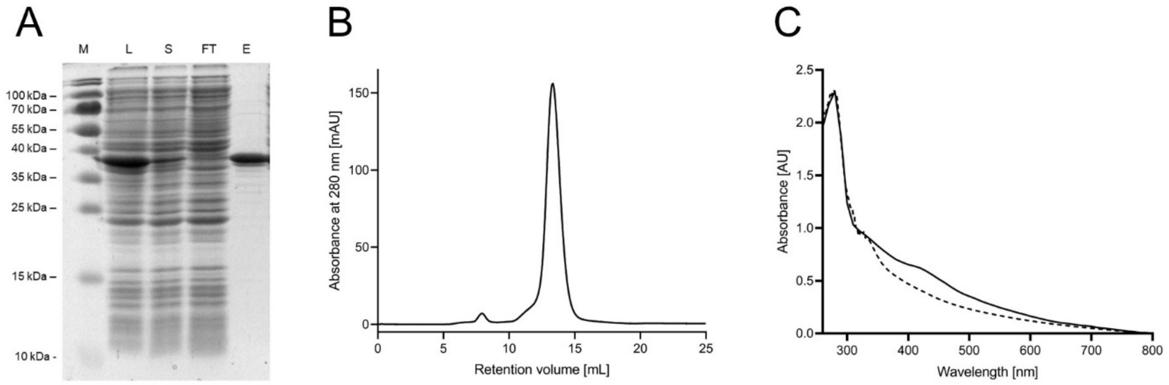

Supplementary Figure S5. A: Recombinant N-6xHis tagged RudS_KT purification using affinity chromatography (M – molecular marker, L – bacterial lysate, S – soluble fraction, FT-flow through, E – eluted protein; theoretical mass 39.21 kDa). B: Elution peak of purified RudS_KT after Superose 12 10/300 GL gel filtration chromatography (mass 37.1 ± 0.7 kDa). C: bold graph – UV-vis spectra of RudS_KT as purified (35 μM); dotted graph – RudS_KT after iron-sulfur cluster reduction with 10 mM sodium dithionite.

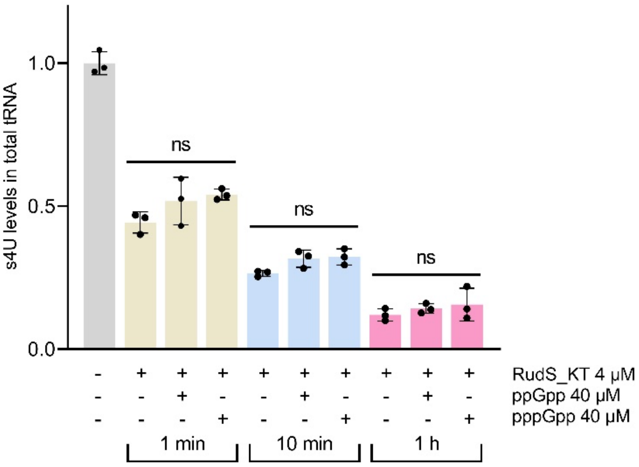

Supplementary Figure S6. Effect of guanosine tetraphosphate and pentaphosphate (ppGpp and pppGpp respectively) on RudS_KT in vitro activity. Three samples obtained within a single time point were compared to each other using one way ANOVA with Bonferroni's post hoc test (n.s.: $p \geqslant 0.05$).

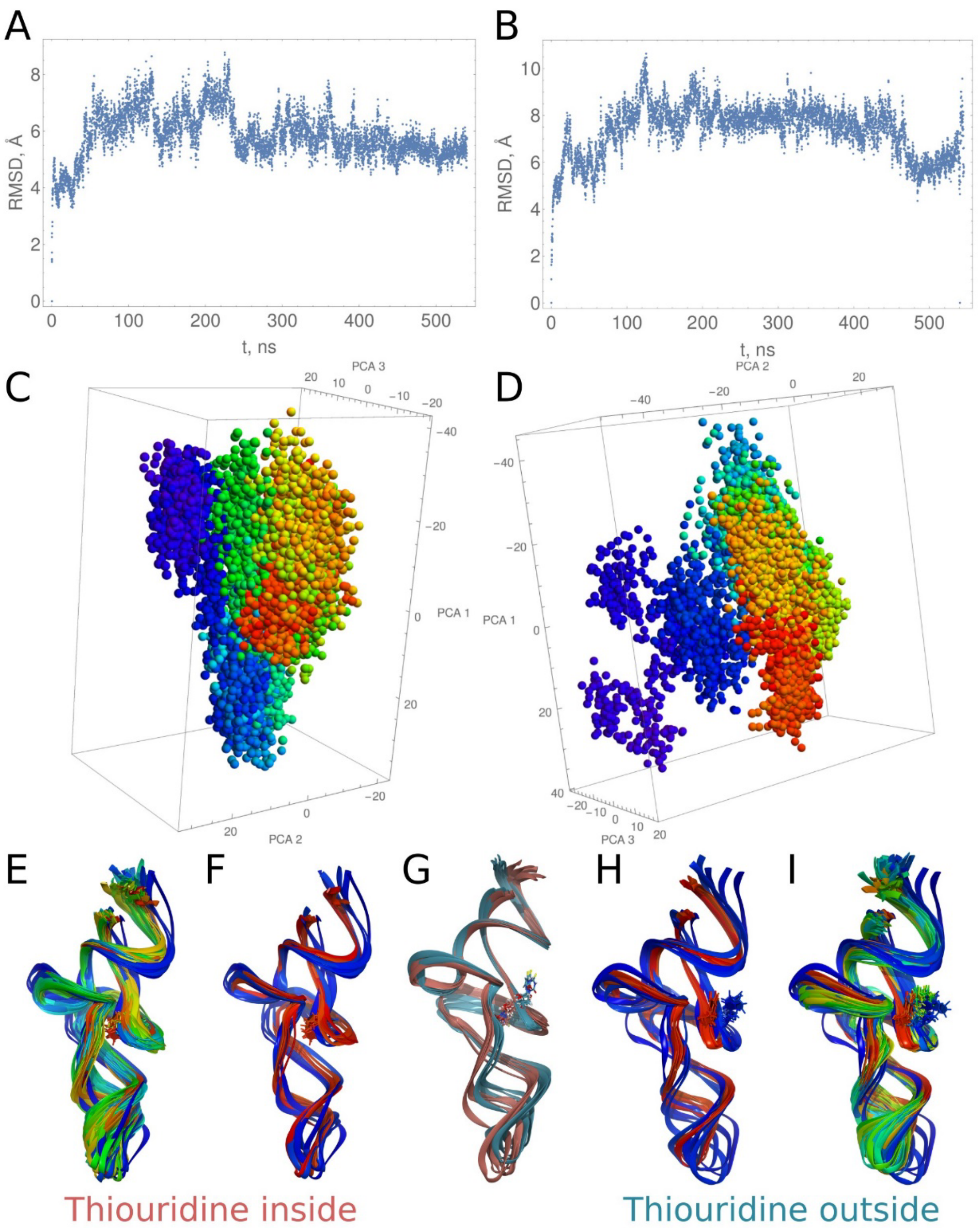

Supplementary Figure S7. Molecular dynamics simulation of phenylalanine tRNA from *E. coli* (PDBID:6y3g). A and B: RMSD plot of MD simulations of native and flipped out thiouridine harboring tRNAs. C and D: PCA analysis of MD simulations of native and flipped out thiouridine harboring tRNAE and I: overall motion of respective tRNAs from MD start (blue) to the end(red). F and H: overall motion of respective tRNAs from MD start (blue) to the end(red) with intermediate frames removed. G: superposition of final MD frames of native and flipped out thiouridine harboring tRNA.

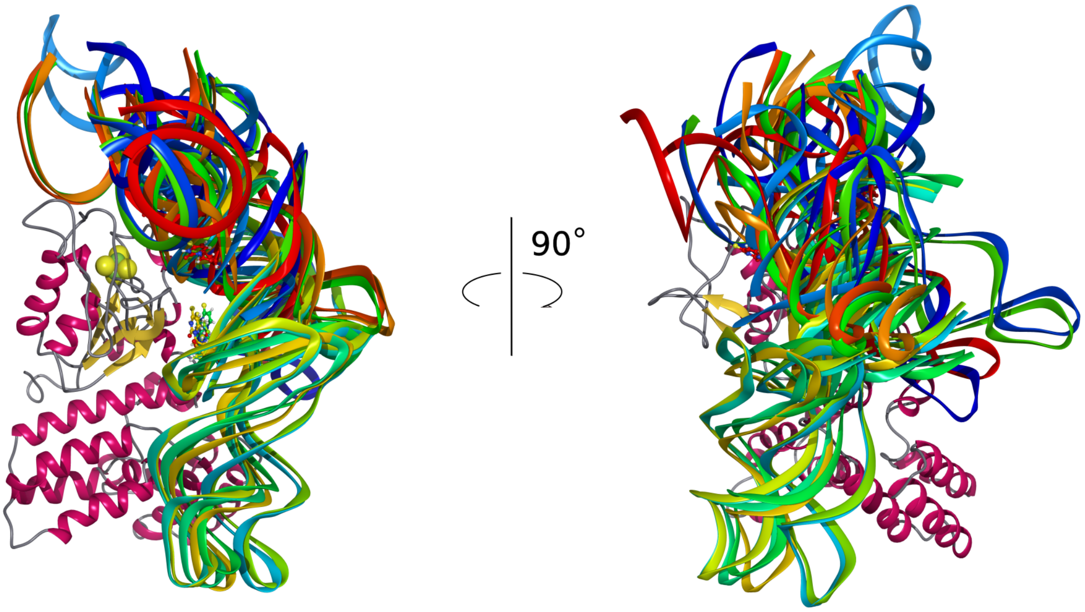

Supplementary Figure S8. Top 15 complexes produced by docking tRNA with thiouridine flipped out to the holoenzyme with HDOCK.

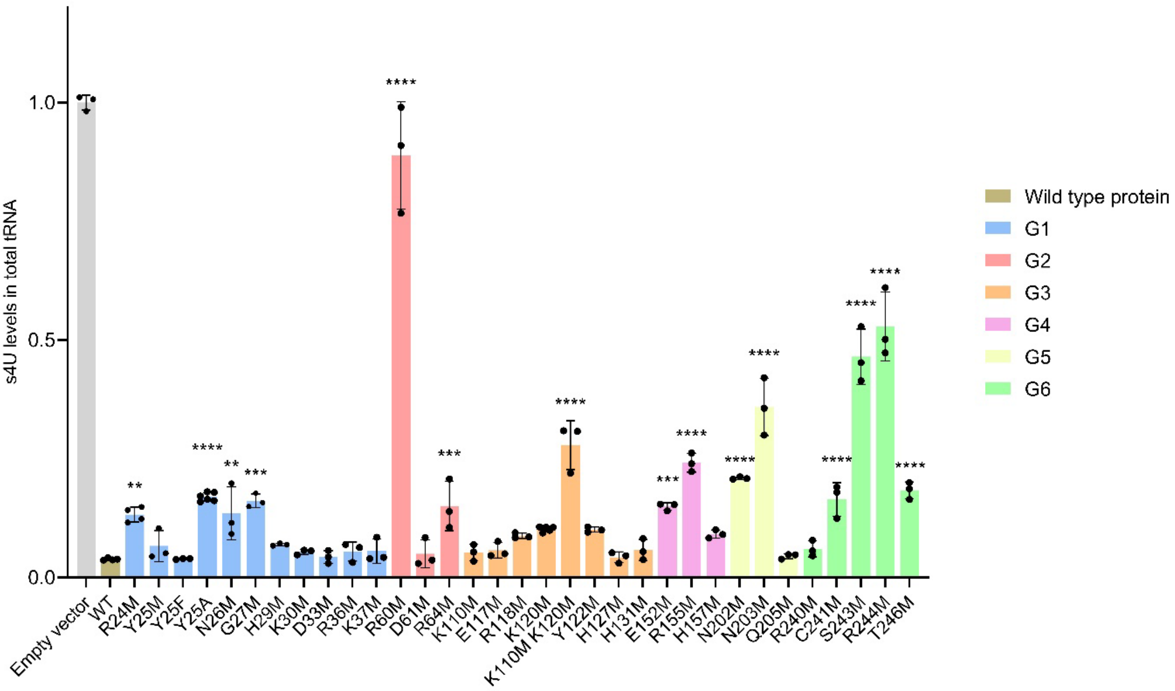

Supplementary Figure S9. s4U levels in E. coli total tRNA upon expression of RudS_KT with single amino acid substitutions. ****: p<0.0001, ***: p<0.001, **: p<0.01, *: p<0.05, n.s.: p⩾0.05 compared to WT, one way ANOVA with Dunnet's post hoc test.

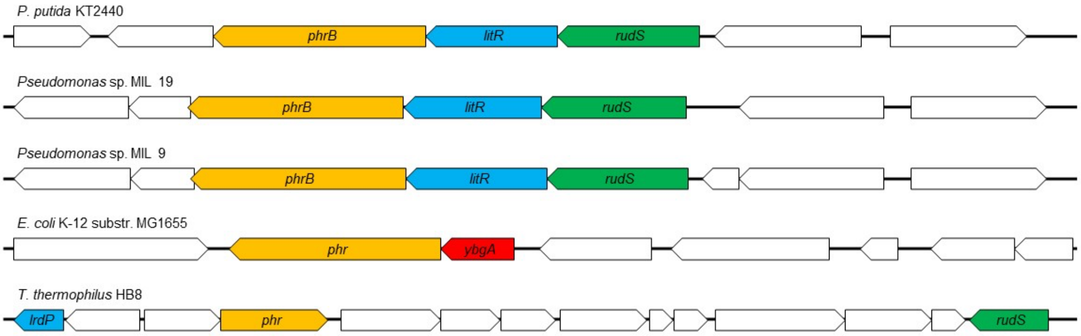

Supplementary Figure S10. Organization of RudS and YbgA encoding genome loci. Genes highlighted in green – RudS encoding gene; red – DUF1722 encoding gene; orange – DNA photolyase encoding genes; blue – light-inducible transcription regulator encoding genes.

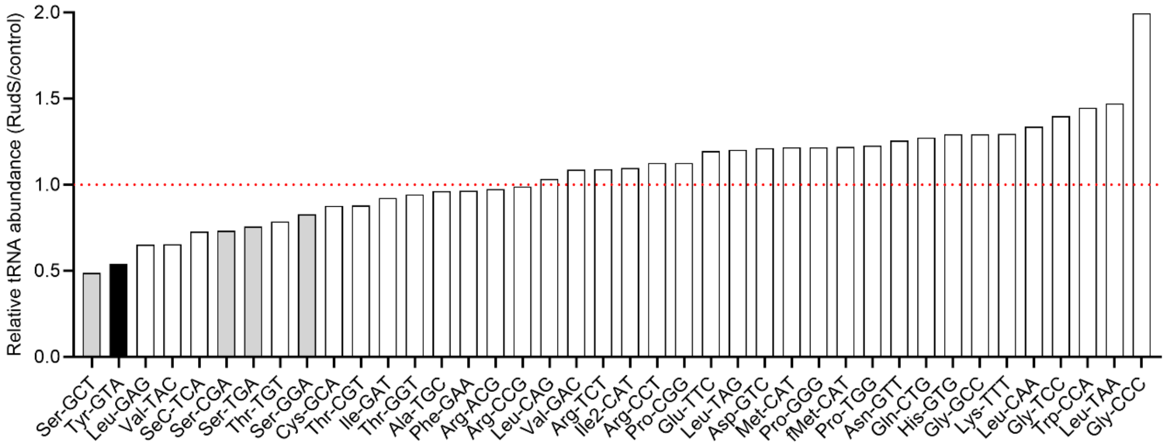

Supplementary Figure S11. Relative tRNA abundance in RudS_KT overexpressing *E. coli* BL21 (DE3) compared to control (empty vector carrying) samples. Black: tyrosine tRNA; grey: serine tRNA's.

REFERENCES

1. Petkevičius,V., Vaitekūnas,J., Gasparavičiūtė,R., Tauraitė,D. and Meškys,R. (2021) An efficient and regioselective biocatalytic synthesis of aromatic N-oxides by using a soluble di-iron monooxygenase PmlABCDEF produced in the Pseudomonas species. *Microb. Biotechnol.*, **14**, 1771–1783.

2. Fuchs,J., Jamontas,R., Hoock,M.H., Oltmanns,J., Golinelli-Pimpaneau,B., Schünemann,V., Pierik,A.J., Meškys,R., Aučynaitė,A. and Boll,M. (2023) TudS desulfidases recycle 4-thiouridine-5′-monophosphate at a catalytic [4Fe-4S] cluster. *Commun. Biol.*, **6**, 1–11.
